# Supplementary material for: The causal relationship between the human gut microbiota and pyogenic arthritis: a Mendelian randomization study
Source: Front Cell Infect Microbiol. 2024 Nov 26;14:1452480. doi: 10.3389/fcimb.2024.1452480 (PMC11629706; doi:10.3389/fcimb.2024.1452480)
Supplement: Supplementary file 1 [file DataSheet1.zip › mendelian test/ebi-a-GCST90027838.csv_forest.pdf]

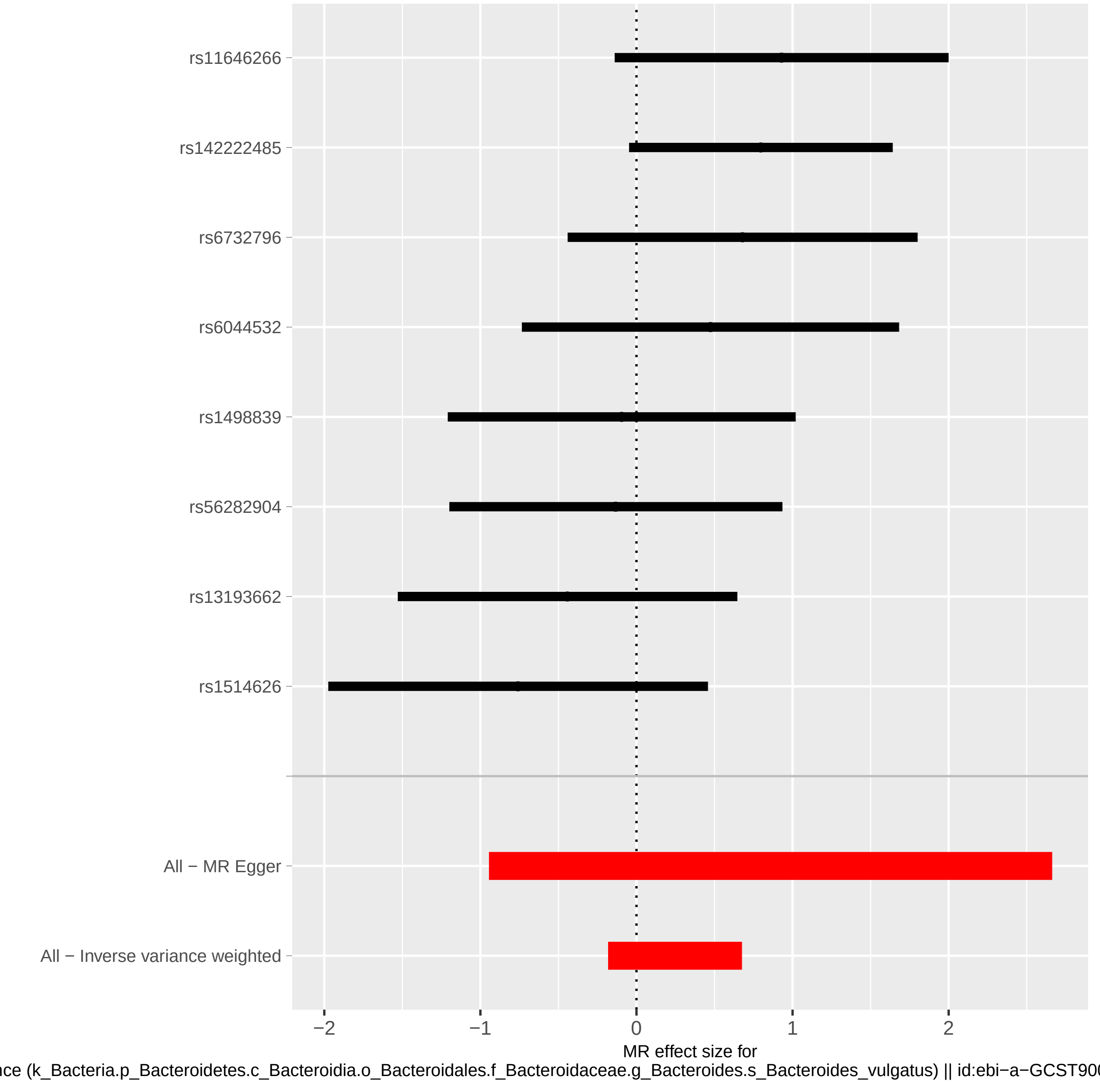
rs11646266
rs142222485
rs6732796
rs6044532
rs1498839
rs56282904
rs13193662
rs1514626
All − MR Egger
All − Inverse variance weighted
−2
−1
0
1
2
MR effect size for
nce (k_Bacteria.p_Bacteroidetes.c_Bacteroidia.o_Bacteroidales.f_Bacteroidaceae.g_Bacteroides.s_Bacteroides_vulgatus) || id:ebi−a−GCST90
